# Knowledge of PrEP Among Healthcare Workers in Substance Use Disorder Services: A Cross-Sectional Study in Italy

**DOI:** 10.1007/s10900-025-01445-x

**Published:** 2025-02-09

**Authors:** Giuseppina Lo Moro, Lorenzo Rosset, Maria Grazia Varì, Alfio Lucchini, Roberta Balestra, Giacomo Scaioli, Roberta Siliquini, Fabrizio Bert

**Affiliations:** 1 Department of Public Health and Paediatric Sciences, University of Turin, Turin, 10126 Italy; 2 Federazione Italiana degli Operatori dei Dipartimenti e dei Servizi delle Dipendenze, (FeDerSerD: Italian Federation of Workers of the Addiction Departments and Services), Milan, Italy; 3 A.O.U. City of Health and Science of Turin, Turin, Italy

**Keywords:** Pre-exposure prophylaxis, HIV, Substance use disorder, Addiction, Healthcare workers

## Abstract

Pre-exposure prophylaxis (PrEP) is an evidence-based strategy for HIV prevention, particularly for high-risk populations such as people who inject drugs and engage in chemsex. In Italy, there is no data on the actual knowledge of PrEP among Healthcare professionals (HCPs) in substance use disorder services (SerDs). This study aimed to assess PrEP awareness among SerD HCPs, also exploring their level of knowledge, practice, training, and perceived barriers. A cross-sectional study was conducted using a convenience sample of HCPs from SerDs across Italy (2023–2024). The questionnaire addressed sociodemographic and work-related information, PrEP awareness, knowledge scores (i.e. percentage of correct answers) on when proposing PrEP and reimbursement criteria, practice, and training received. Multiple logistic regression was performed to explore associations with PrEP awareness. The sample consisted of 306 professionals (26.8% physicians). Only 44.8% were aware of PrEP, with lower awareness among non-physicians (*p* < 0.001). As for knowledge scores on when proposing PrEP and on reimbursement, the median was 57.14% (IQR: 42.86–71.43%) and 55.56% (IQR: 33.33–66.67%), respectively. No differences were reported across professional roles. Only 10.36% reported PrEP is offered at their workplace and 87.9% highlighted a lack of training. Additionally, 96.79% believed it would be appropriate for HCPs to receive PrEP training; however, nurses were the most likely to state it is not appropriate (*p* = 0.046). The study identified gaps in PrEP knowledge and training among SerD professionals, including physicians. The strong interest in training suggests that SerD HCPs, though with varying perceptions, may be a receptive group for interventions for improving PrEP implementation.

## Introduction

Pre-exposure prophylaxis (PrEP) is an evidence-based prevention strategy primarily used to reduce the risk of HIV infection [1]. PrEP has been demonstrated to be a safe and effective intervention, particularly for high-risk populations, e.g. men who have sex with men (MSM), serodiscordant couples, and people who inject drugs [2, 3]. International organizations, such as the Joint United Nations Programme on HIV/AIDS (UNAIDS) and the World Health Organization (WHO), have developed comprehensive strategies and guidelines [4–6] for the use of antiretroviral drugs in both the treatment and prevention of HIV infection, emphasizing the importance of making PrEP accessible to those at the highest risk and integrating it into broader HIV prevention and care strategies.

PrEP involves the preventive use of antiretroviral medications, specifically tenofovir disoproxil fumarate and emtricitabine [1]. In Italy, these medications are included among the restricted prescription drugs by the Italian Medicines Agency (AIFA), which are available only through infectious disease specialists or in hospital settings, reflecting the need for specialized knowledge and monitoring associated with its use [7]. The Italian Society of Infectious and Tropical Diseases (SIMIT) and the Italian Ministry of Health have issued guidelines prioritizing PrEP for individuals at high risk of HIV infection [8]. These guidelines provide specific criteria for PrEP eligibility to ensure that those who can benefit most from the intervention have access to it, i.e. MSM, transgender women, and heterosexuals with certain high-risk behaviours in the past three months; people with injection drug use history in the past 6 months and with ongoing treatment in specialized centres; and sexual or injecting partners of HIV-positive individuals not on treatment [8].

Furthermore, the recent decision made by AIFA in 2023 marked an important step in the fight against HIV in Italy [7], as it approves the reimbursement of PrEP by the National Health Service for some categories of at-risk individuals. This decision aligns Italy with the recommendations of other European countries, promoting PrEP as a key preventive option for HIV [9]. Eligibility criteria include being over 18, a negative HIV test, and high-risk sexual behaviour within the past three months, such as unprotected sex with HIV-positive or unknown-status partners, treatment for sexual transmitted infections (STI), prior post-exposure prophylaxis (PEP) use, or use of drugs during sexual activities (chemsex) [7]. Regarding chemsex, it has been reported that sexualized drug use correlates with moderate to high risk for substance use disorder [10]. Addressing chemsex within addiction services may be crucial for combining HIV prevention with substance use interventions, promoting a holistic approach.

It is worth noting that healthcare professionals (HCPs) may play a crucial role in PrEP implementation. Studies conducted in diverse populations and healthcare settings have highlighted substantial barriers related to the role of healthcare providers. For instance, several authors reported missed opportunities for PrEP implementation, with gaps in discussions by general practitioners [11] and primary care physicians [12], and with many newly diagnosed HIV patients lacking documented sexual history or PrEP counseling [13]. Vincent and McFarland [14] further highlighted that healthcare providers frequently missed opportunities to discuss PrEP with people who inject drugs, despite their interaction with healthcare services. The importance of engaging addiction services is underscored by Rozansky et al. [15], who reported that patients seen by addiction consult services in hospital were more likely to receive PrEP. Together, these findings show the critical need to involve a broader spectrum of healthcare professionals, including those in substance use care, to optimize PrEP delivery and uptake.

In Italy, specialized addiction services, such as SerD (*Servizi per le Dipendenze patologiche*, i.e. services for substance use disorders and addictive disorders) facilities, are uniquely positioned to address these gaps. SerDs are public facilities within the Italian National Health Service, with over 550 SerD active throughout Italy. These facilities engage in activities related to prevention, diagnosis, treatment, rehabilitation, and social and occupational reintegration of individuals with substance use disorders, often in collaboration with therapeutic communities [16]. SerD professionals, including nurses, doctors, psychologists, social workers, and educators, are in a privileged position to identify individuals at high risk of HIV infection and to propose PrEP as a preventive measure. However, due to the current lack of data, the level of knowledge on PrEP among SerD healthcare workers in Italy remains unclear, potentially leading to gaps in understanding eligibility criteria and promoting this preventive strategy to suitable patients.

Therefore, our study primarily aimed to assess knowledge regarding PrEP among Italian healthcare professionals working in SerD facilities, particularly in light of recent updates on reimbursement of the treatment, and to explore potential sociodemographic and work-related characteristics associated with this knowledge. A secondary aim was to evaluate how well these professionals understand PrEP recommendation and reimbursement criteria, the frequency with which PrEP is proposed in their clinical practice, their perceptions of the training they receive on this topic, and the barriers they perceive in implementing PrEP. The findings from this study may provide valuable insights into the current state of PrEP implementation in SerD, highlighting areas for improvement to enhance the effectiveness of HIV prevention efforts in this context.

## Materials and methods

### Study Design

A cross-sectional study was conducted on a convenience sample of healthcare professionals working in SerD (services for substance use disorders and addictive disorders) in Italy. Participants were required to be nurses, doctors, psychologists, social workers, professional educators, and other professionals actively involved in patient care and assistance. Non-SerD workers and administrative personnel were excluded. The study protocol received approval from the Bioethics Committee of the University of Turin (Protocol number 0623774, 23 November 2023). Participation was voluntary and anonymous, with no compensation provided to participants.

The questionnaire was distributed between November 2023 and May 2024 using three approaches: researchers shared link on major social media platforms (mainly WhatsApp); the main Italian organization representing professionals from Departments and Services for substance use disorders and addictive disorders nationwide (FeDerSerD) disseminated the link among its members; and all SerD facilities across Italy were contacted by email.

### Questionnaire

The self-administered questionnaire was set on the online platform LimeSurvey (https://www.limesurvey.org/). Before accessing the questionnaire, participants were required to accept the informed consent form. The questionnaire was structured into three main sections. Multiple-choice or single-choice questions were predominantly used.

In the first section, socio-demographic data such as gender, age, educational level, citizenship, and region of residence were collected. Participants were asked to confirm whether they were currently working in a SerD and to indicate their current professional role. If participants were not employed in a SerD, they terminated the questionnaire at this point.

The second section explored knowledge about PrEP. Participants were asked if they knew what PrEP is (primary outcome “PrEP awareness”). For negative responses, participants were directed to the third section. For affirmative responses, the second section delved into participants’ knowledge. Participants were asked how they acquired such knowledge, in which situations PrEP may be recommended (with various possible prescription scenarios presented [17]), which healthcare professional is authorized to prescribe PrEP [17], and whether PrEP is reimbursed by the National Health Service [7]. If participants believed PrEP is reimbursable, specific conditions required for reimbursement were investigated, based on the latest AIFA indications [17]. Knowledge scores were calculated for both possible prescription scenarios and reimbursement criteria, assigning one point per correct answer and determining the percentage of correct responses for each.

The third section, which was mostly related to participants’ SerD experience, began with an explanation on what PrEP is, when it is recommended, and to whom [6]. Subsequently, the questionnaire assessed the perception of PrEP as a valid HIV prevention strategy. Then, participants were asked if in their practical experience PrEP was proposed to patients in their services. If PrEP had been proposed in their SerD, the frequency of its prescription during the last year and participants’ perceptions regarding its appropriate recommendation were explored. Difficulties encountered in prescribing PrEP, such as challenges in finding a prescribing physician or communication difficulties with clinics for STI, were also investigated. Finally, participants were asked if training on PrEP had ever been offered to them, their perception of their competence in this regard, and their opinion on the appropriateness of healthcare professionals receiving training on PrEP.

### Statistical Analysis

A descriptive analysis was performed for all variables. The Shapiro-Wilk test for normality was conducted for age and knowledge scores for possible prescription scenarios and reimbursement criteria. Since none of these variables followed a normal distribution, they were described using medians and interquartile ranges (IQRs).

To verify whether there were differences between those who completed the questionnaire and those who did not finish the third section, a Mann-Whitney U test was performed for age, and chi-square tests were conducted for gender, professional role, degree, and PrEP awareness.

Considering the overall sample, PrEP awareness was the primary outcome. Chi-square tests (Mann Whitney U test for age) were conducted to describe any differences in sociodemographic, work-related, and SerD experience variables between those who were familiar with PrEP and those who were not. A multiple logistic regression model was conducted to explore the influence of sociodemographic and occupational variables on the likelihood of knowing PrEP. The results were presented as adjusted Odds Ratios (adjOR) with 95% confidence intervals (CI).

Additionally, a chi-square test was conducted to assess a possible association between professional role and the opinion regarding the appropriateness of PrEP training for healthcare professionals.

For the subsample reporting PrEP awareness, regarding both knowledge scores, Spearman correlation tests were performed to assess potential correlations with age, and Kruskal-Wallis tests were used to examine the distribution of such scores across professional roles. Similarly, Mann-Whitney tests (for age) and chi-square tests (for professional roles) were conducted to analyse differences in knowledge about who can prescribe PrEP and whether PrEP can be reimbursed under certain conditions.

Statistical analysis was carried out using SPSS (v29) and Stata (v18), with a two-tailed p-value < 0.05 considered significant. Missing values were excluded.

## Results

### Overall Sample

The overall sample consisted of 306 participants who completed at least the socio-demographic characteristics and the primary outcome “PrEP awareness”. A total of 8% of the sample did not complete the third section. No significant differences were reported between participants who completed the entire questionnaire (*n* = 280) and those who did not complete the third section (*n* = 26) (gender: *p* = 0.844; age: *p* = 0.735; professional role: 0.540; degree: *p* = 0.531; PrEP awareness: *p* = 0.882).

Table 1 shows the characteristics of the sample. The sample consisted entirely of Italian citizens, with 66.3% from the northern regions of the country. The median age was 52 years (IQR: 45–59 years). The most represented occupations were non-infectious disease doctors (22.2%), nurses (23.2%), and psychologists (21.2%).

**Table 1:** Characteristics of the sample and associations with the primary outcome “PrEP awareness”

Variable	Overall sample	Pre-exposure prophylaxis (PrEP) awareness		
	*n* = 306 *N* (%)	No (*n* = 169) *N* (%)	Yes (*n* = 137) *N* (%)	*p*-value
**Gender**				
Female	241 (79.28)	140 (58.09)	101 (41.91)	0.052
Male	63 (20.72)	28 (44.44)	35 (55.56)	
**Degree**				
No	46 (15.03)	27 (58.7)	19 (41.3)	0.608
Yes	260 (84.97)	142 (54.62)	118 (45.38)	
**Geographical area**				
Northern Italy	203 (66.34)	106 (52.22)	97 (47.78)	0.330
Central Italy	46 (15.03)	28 (60.87)	18 (39.13)	
Southern Italy and Islands	57 (18.63)	35 (61.40)	22 (38.60)	
**Professional role**				
Physician (except infectious disease specialist)	68 (22.22)	23 (33.82)	45 (66.18)	**< 0.001**
Infectious disease specialist	14 (4.58)	0 (0)	14 (100)	
Nurse	71 (23.2)	40 (56.34)	31 (43.66)	
Psychologist	65 (21.24)	43 (66.15)	22 (33.85)	
Social worker	37 (12.09)	29 (78.38)	8 (21.62)	
Professional educator	37 (12.09)	23 (62.16)	14 (37.84)	
Other (e.g. psychiatric rehabilitation technician)	14 (4.58)	11 (78.57)	3 (21.43)	

Figures are expressed as frequencies and percentages in brackets (column percentages in the “Overall sample” column and row percentages in the “PrEP awareness” columns). P-value obtained via chi squared test

Regarding our primary outcome, only 44.8% of participants were aware of PrEP (*n* = 137). Neither age (*p* = 0.100) nor other socio-demographic variables were associated with PrEP awareness (Table 1). The professional role was significantly associated with PrEP awareness: the highest percentages were reported among non-infectious disease physicians (66.18%) and infectious disease specialists (100%).

After respondents were informed about PrEP (third section of the questionnaire) (Table 2), over half of the sample (55.36%) strongly thought that PrEP was a valid preventive strategy (no participant selected “not at all”). Only 10.36% of participants reported that PrEP is offered at their workplace. Among them, most were either unaware of how often PrEP is offered (*n* = 12, 41.38%) or indicated that it is recommended between 1 and 5 times per year (*n* = 11, 37.93%). Slightly more than half believed that PrEP is offered adequately (*n* = 16, 55.17%), while the remaining participants felt that PrEP is offered less frequently than would be appropriate (*n* = 13, 44.83%).

**Table 2:** Main items from the third section and associations with the primary outcome “PrEP awareness”

Variable	Overall sample	Pre-exposure prophylaxis (PrEP) awareness		
	*n* = 280 *N* (%)	No (*n* = 169) *N* (%)	Yes (*n* = 137) *N* (%)	*p*-value
**Perceiving PrEp as a valid preventive strategy**				
Slightly	24 (8.57)	20 (83.33)	4 (16.67)	**< 0.001**
Moderately	101 (36.07)	68 (67.33)	33 (32.67)	
Very	155 (55.36)	67 (43.23)	88 (56.77)	
**PrEP is offered in their facility**				
Yes	29 (10.36)	4 (13.79)	25 (86.21)	**< 0.001**
No	175 (62.5)	104 (59.43)	71 (40.57)	
Participant does not know	76 (27.14)	47 (61.84)	29 (38.16)	
**Training on PrEP had ever been offered**				
Yes	11 (3.93)	2 (18.18)	9 (81.82)	**0.009**
No	246 (87.86)	136 (55.28)	110 (44.72)	
Participant does not know	23 (8.21)	17 (73.91)	6 (26.09)	
**Self-perceived level of competence regarding PrEP**				
Not at all	146 (52.14)	121 (82.88)	25 (17.12)	**< 0.001**
Slightly	114 (40.71)	30 (26.32)	84 (73.68)	
Moderately	19 (6.79)	4 (21.05)	15 (78.95)	
Very	1 (0.36)	0 (0)	1 (100)	
**Thinking it is appropriate for healthcare workers to receive training on PrEP**				
No	9 (3.21)	8 (88.89)	1 (11.11)	**0.040**
Yes	271 (96.79)	147 (54.24)	124 (45.76)	

Figures are expressed as frequencies and percentages in brackets (column percentages in the “Overall sample” column and row percentages in the “PrEP awareness” columns). P-value obtained via chi squared test

Exploring potential major difficulties in prescribing PrEP, 60 participants reported a lack of experience (21.43%), 23 the need for more information on the topic (8.21%), 57 difficulty in finding a doctor who can prescribe PrEP (20.36%), 64 challenging communication with STI clinics (22.86%), and 5 poor patient compliance (1.79%) (multiple responses could be selected).

Furthermore, 87.9% reported a lack of specific training on PrEP for healthcare professionals in their facilities. Most participants perceived themselves as having no competence at all (52.14%) or only slight competence (40.71%) regarding PrEP and 96.79% believed it would be appropriate for healthcare workers to receive training on PrEP. A difference was observed in the opinion regarding the appropriateness of training for healthcare professionals: among professional roles, nurses were the most likely to state that it is not appropriate (9.38%) (*p* = 0.046).

Table 2 shows the relationships between the main items of the third section and PrEP awareness. Overall, participants who reported knowing what PrEP is had significantly more experience and training on the topic developed within their SerD.

Lastly, exploring the influence of sociodemographic and occupational variables on the likelihood of knowing PrEP, the multiple logistic regression model showed that all professional roles had lower odds of reporting awareness compared with physicians (Table 3).

**Table 3:** Multiple logistic regression model with outcome “PrEp awareness”

Variable	adjOR 95% CI	*p*-value
**Age**	1.01 (0.98–1.03)	0.708
**Gender**		
Female	Ref.	
Male	1.23 (0.66–2.30)	0.519
**Geographical area**		
Northern Italy	Ref.	
Central Italy	0.75 (0.37–1.53)	0.435
Southern Italy and Islands	0.62 (0.32–1.21)	0.163
**Degree**		
No	Ref.	
Yes	0.95 (0.41–2.20)	0.908
**Professional role**		
Physician (except infectious disease specialist)	Ref.	
Infectious disease specialist	-*	
Nurse	0.36 (0.16–0.82)	**0.015**
Psychologist	0.28 (0.13–0.59)	**< 0.001**
Social worker	0.14 (0.05–0.37)	**< 0.001**
Professional educator	0.3 (0.13–0.71)	**0.006**
Other (e.g. psychiatric rehabilitation technician)	0.13 (0.03–0.56)	**0.006**

*omitted (all 14 infectious disease specialists knew PrEP)

### Subsample Reporting PrEP Awareness

Among the 137 participants who knew PrEP, the majority (89.05%) learned about PrEP for work-related reasons, while 5.11% discovered it through the internet, 2.92% through personal connections, and 2.92% selected “other.” In the scenarios presented for possible PrEP prescription (which included both true and false statements), participants provided incorrect responses in the following cases: 90.51% pregnancy and breastfeeding, 70.07% recent STI diagnosis or treatment, 43.80% injection drug use, 24.82% history of recent high-risk sexual encounters, 16.06% partner positive for HIV, 12.41% ongoing PEP, and 75.18% family members with HIV positivity. The median knowledge score for the scenarios in which PrEP can be prescribed was 57.14% (IQR: 42.86–71.43%). No significant differences in distribution were reported across professional roles (*p* = 0.704), and no correlation with age was observed (*p* = 0.635).

Furthermore, 77.37% correctly indicated that only the infectious disease specialist can prescribe PrEP (no association with professional role, *p* = 0.482, or age, *p* = 0.242), and 30.66% knew that PrEP can be reimbursed by the National Health Service under some conditions (no association with professional role, *p* = 0.332, or age, *p* = 0.971). In contrast, 43.07% stated that PrEP can always be reimbursed, 2.19% believed it cannot be reimbursed, and 24.09% reported not knowing. Among participants that stated that PrEP can be reimbursed, the frequency of incorrect responses regarding the criteria for reimbursement (which included both true and false statements) was: 64.21% age over 18, 74.74% previous PEP use, 43.16% individuals with recent HIV exposure (< 1 month), 60.00% negative HIV Ab/Ag test, 25.26% high-risk sexual behaviour for HIV acquisition in the past 3 months, 71.58% ongoing STI treatment, 52.63% chemsex, 14.74% individuals with HIV infection, and 32.63% for having had at least one unprotected sexual encounter with an HIV-positive or status-unknown casual partner. The median knowledge score for the reimbursement criteria was 55.56% (IQR: 33.33–66.67%). No significant differences in distribution were reported across professional roles (*p* = 0.070), and no correlation with age was observed (*p* = 0.175).

## Discussion

The primary objective of this study was to assess the awareness of PrEP among healthcare professionals working in SerD facilities and to identify associated sociodemographic and work-related factors. Secondarily, it aimed to explore participants’ understanding of the criteria for recommending PrEP and its reimbursement, as well as to examine their experiences with PrEP in practice and the barriers they may face in its implementation.

First of all, our study highlighted that slightly less than half of the participants were aware of what PrEP is. While direct comparisons are limited due to the lack of similar studies in equivalent settings, it is worth noting that Navarro-Aguilar et al. reported higher awareness levels (69%) in emergency care professionals in Spain [18]. This alarming low level of PrEP awareness in our sample raises concerns, especially considering the critical role of professionals working with individuals with substance use disorders [14, 15], and underscores the need to critically evaluate whether the current level of knowledge among these professionals is sufficient to ensure adequate access to this preventive intervention. Notably, the age of professionals did not appear to influence PrEP knowledge, suggesting that gaps are more likely attributable to discrepancies in training practices and the dissemination of information rather than generational differences. Physicians demonstrated higher awareness compared with other groups, even when adjusting for other variables in multiple regression analyses. However, it is concerning that only 66% of physicians (excluding infectious disease specialists) were aware of PrEP. This highlights the need to enhance training not only for non-medical staff but also for physicians. Supporting this observation, our subsample analysis of those reporting PrEP awareness revealed limited knowledge of specific details about PrEP, with no significant differences across professional roles. Similar trends have been observed in other contexts involving substance use disorders services, where physicians exhibited higher awareness but lack detailed knowledge [19]. Our questions did not delve deeply into the mechanisms of PrEP but focused on essential information to identify candidates for PrEP referral to infectious disease specialists. This lack of knowledge, even among those aware of PrEP, could hinder patient access to this preventive measure. Finally, recognizing the low knowledge on PrEP, it is essential to consider that interventions should not only aim to enhance awareness but also focus on improving provider comfort and skills in HIV risk assessment. These have been identified as suboptimal in other settings [20] and warrant further investigation within our context.

The results from the third section, which was mostly on the experience of participants within their services, highlighted several issues regarding the integration of PrEP in SerD facilities. While a small proportion of participants (8.57%) perceived PrEP as only slightly valid, the remaining participants recognized its substantial potential, suggesting that SerD healthcare professionals may be a highly receptive group for targeted interventions aimed at enhancing training on the topic. However, the fact that only 10.36% of participants reported that PrEP is recommended at their workplace pointed to a significant gap in its actual implementation. This lack of availability cannot be solely attributed to the limited awareness among professionals but is likely influenced by a combination of factors, including insufficient training, lack of institutional support, and the disparity in access to PrEP among the various centers, which should be further investigated.

Indeed, the lack of specific training on PrEP for healthcare professionals, reported by 87.9% of participants, underscored the challenges to its effective implementation, compounded by the fact that over 90% of participants felt they had little or no competence regarding PrEP. Despite this, a large majority (96.79%) acknowledged the appropriateness of PrEP training for their professional roles, highlighting not only a clear need for targeted educational initiatives but also an interest in receiving such training. Interestingly, nurses were more likely than other professionals to disagree on the appropriateness of PrEP training, suggesting a potential difference in how relevant they perceive PrEP training to be for their specific roles. This raises the need to reflect on the development of role-specific interventions to address the varying perceptions and educational needs across different professional groups. Moreover, participants who reported knowing what PrEP is had more experience and training on the topic within their SerD, indicating that familiarity with PrEP is closely linked to prior exposure and institutional training opportunities, thus highlighting the need for institutional efforts to provide comprehensive training and integrate PrEP recommendation into routine care. Supporting this, Blumenthal et al. [21] showed that greater knowledge is associated with increased acceptance and willingness to prescribe PrEP, emphasizing that adequate training, even during professional education, can enhance healthcare professionals’ competence in proposing PrEP and boost their confidence. Interestingly, a recent literature review on PrEP training offerings in the USA in medical schools and subsequent courses highlighted that HIV PrEP training is often inadequate and inconsistent [22]. Additionally, a U.S. study by Cooper et al. [23] revealed that only 38% of medical schools included content on PrEP. Although similar studies have not been conducted in Italy, these insights point to the need of incorporating this topic into the education of future healthcare professionals. Educational programs aimed at both students and professionals could play a crucial role in addressing this gap, as they have been shown to improve knowledge and awareness of PrEP [24, 25].

Finally, our findings highlighted several barriers to PrEP prescription in our sample. The most frequently reported challenges included structural and organizational issues, such as difficulties in communicating with STI clinics and finding a doctor who can prescribe PrEP, which may indicate a potential gap in healthcare accessibility and provider availability. Similar organizational barriers have been documented in other contexts, including the rapid implementation of PrEP programs without sufficient resources or staff [26, 27] and the limited time available to discuss PrEP [27].

On the other hand, training-related issues also emerged, such as a lack of experience and the need for more information on the topic, reflecting an ongoing demand for educational initiatives to enhance knowledge and confidence among healthcare professionals. Previous studies have identified key barriers such as limited awareness and knowledge about PrEP [28], lack of familiarity with prescribing protocols [26], and discomfort with providing PrEP counseling [27].

Addressing these challenges may require a multifaceted approach, including improved communication pathways between services, expanded training programs, and a more supportive environment for PrEP implementation. Potential facilitators identified in the literature include leadership engagement, integration into existing workflows, alignment with holistic care principles, the application of multi-level interventions, inclusion of non-medical staff perspectives [27], and opportunities to acquire practice-based knowledge [26].

This study had several limitations. The sample size was limited, and the distribution of respondents was not well-balanced across Italian regions, thus limiting the generalizability of the findings. Additionally, participants needed to have access to a computer or mobile device to respond to the online survey, which, despite being simple, may have excluded some healthcare professionals who do not regularly use these technologies. Despite its limitations, this work represents one of the first Italian studies addressing the topic of PrEP within this specific setting. It provides a valuable starting point for introducing and contextualizing this issue, laying the groundwork for further research and targeted interventions.

## Conclusions

This study offered initial insights into PrEP awareness among SerD healthcare professionals in Italy, revealing significant gaps in knowledge and training, even among physicians. The findings highlighted the need for targeted initiatives to improve awareness and competence, addressing both individual and systemic barriers. Despite the limited availability of PrEP in workplaces, the strong interest in training suggests that SerD professionals, though with varying perceptions based on their professional roles, may be a receptive group for interventions aimed at improving the implementation and accessibility of this preventive measure.

## Data Availability

The datasets generated during and/or analysed during the current study are available from the corresponding author on reasonable request.

## References

[1] Davies, O., Ustianowski, A., & Fox, J. (2016). Pre-exposure Prophylaxis for HIV Prevention: Why, what, who and how. *Infectious Diseases and Therapy*, *5*(4), 407–416. 10.1007/s40121-016-0128-8 27677264 10.1007/s40121-016-0128-8 PMC5125131

[2] Traeger, M. W., Schroeder, S. E., Wright, E. J., Hellard, M. E., Cornelisse, V. J., Doyle, J. S., & Stoové, M. A. (2018). Effects of Pre-exposure Prophylaxis for the Prevention of Human Immunodeficiency Virus infection on sexual risk behavior in men who have sex with men: A systematic review and Meta-analysis. *Clinical Infectious Diseases*, *67*(5), 676–686. 10.1093/cid/ciy182 29509889 10.1093/cid/ciy182

[3] Murchu, O., Marshall, E., Teljeur, L., Harrington, C., Hayes, P., Moran, C., P., & Ryan, M. (2022). Oral pre-exposure prophylaxis (PrEP) to prevent HIV: A systematic review and meta-analysis of clinical effectiveness, safety, adherence and risk compensation in all populations. *British Medical Journal Open*, *12*(5), e048478. 10.1136/bmjopen-2020-048478 10.1136/bmjopen-2020-048478 PMC9096492 35545381

[4] Joint United Nations Programme on HIV/AIDS (UNAIDS). (2023). *The path that ends AIDS: UNAIDS Global AIDS Update 2023*. Geneva.

[5] Joint United Nations Programme on HIV/AIDS (UNAIDS) (2021). *Global AIDS Strategy 2021–2026. End inequalities. End AIDS.* Geneva.

[6] World Health Organization (WHO) (2016). *Consolidated guidelines on the use of antiretroviral drugs for treating and preventing HIV infection: recommendations for a public health approach, 2nd ed*. 27466667

[7] Agenzia Italiana Del Farmaco (AIFA). Determina 8 maggio 2023 Regime di rimborsabilità e prezzo, a seguito di nuove indicazioni terapeutiche, del medicinale per uso umano «Emtricitabina/Tenofovir Disoproxil Mylan». (Determina n. 349/2023). (23A02836) (GU Serie Generale n.115 del 18-05-2023) (2023).

[8] SIMIT, & Ministero della Salute (2017). *Linee Guida Italiane sull’utilizzo della Terapia Antiretrovirale e la gestione diagnostico-clinica delle persone con infezione da HIV-1*.

[9] ECDC (2023). *Evidence Brief Pre-exposure prophylaxis for HIV prevention in Europe and Central Asia Monitoring implementation of the Dublin Declaration on Partnership to fight HIV/AIDS in Europe and Central Asia– 2022 progress report February 2023*. Stockholm.

[10] Torres, T. S., Bastos, L. S., Kamel, L., Bezerra, D. R. B., Fernandes, N. M., Moreira, R. I., & De Boni, R. B. (2020). Do men who have sex with men who report alcohol and illicit drug use before/during sex (chemsex) present moderate/high risk for substance use disorders? *Drug and Alcohol Dependence*, *209*, 107908. 10.1016/j.drugalcdep.2020.107908 32078972 10.1016/j.drugalcdep.2020.107908

[11] Lions, C., Laroche, H., Mora, M., Pialoux, G., Cotte, L., Cua, E., & Spire, B. (2023). Missed opportunities for < scp > HIV pre-exposure prophylaxis among people with recent < scp > HIV infection: The French < scp > ANRS> 95041 < scp > OMaPrEP study. *HIV Medicine*, *24*(2), 191–201. 10.1111/hiv.13367 35943165 10.1111/hiv.13367

[12] Elopre, L., Ott, C., Lambert, C. C., Amico, K. R., Sullivan, P. S., Marrazzo, J., & Turan, J. M. (2021). Missed Prevention opportunities: Why Young, Black MSM with recent HIV diagnosis did not Access HIV Pre-exposure Prophylaxis services. *AIDS and Behavior*, *25*(5), 1464–1473. 10.1007/s10461-020-02985-0 32749626 10.1007/s10461-020-02985-0 PMC7858694

[13] Lopez, M. G., Alvarez, K. S., Harms, M., Smith, M., Levy Kamugisha, E., Arnold, E. M., & King, H. L. (2024). Missed opportunities for HIV Prevention in a large County Safety Net Health System. *The Journal of the American Board of Family Medicine*, *37*(2), 261–269. 10.3122/jabfm.2023.230297R2 38740488 10.3122/jabfm.2023.230297R2

[14] Vincent, W., & McFarland, W. (2022). Missed opportunities for healthcare providers to discuss HIV preexposure prophylaxis with people who inject drugs. *International Journal of Drug Policy*, *110*, 103873. 10.1016/j.drugpo.2022.103873 36252292 10.1016/j.drugpo.2022.103873

[15] Rozansky, H., Christine, P. J., Younkin, M., Fox, J. M., Weinstein, Z. M., Suarez, S., & Taylor, J. L. (2024). Addiction consult service involvement in PrEP and PEP delivery for patients who inject drugs admitted to an urban essential hospital. *Addiction Science & Clinical Practice*, *19*(1), 77. 10.1186/s13722-024-00502-5 39497126 10.1186/s13722-024-00502-5 PMC11533369

[16] Governo Italiano. (n.d.). Ser.D. Retrieved December 12 (2024). from https://www.politicheantidroga.gov.it/it/servizi-sul-territorio/serd/

[17] Agenzia Italiana Del Farmaco (AIFA). Determina 7 Agosto 2023 - Aggiornamento della scheda di prescrizione cartacea per l’associazione emtricitabina / tenofovir disoproxil Mylan/EG/Teva nella profilassi pre-esposizione (PrEP). (Determina n. DG/323/2023). (23A04846) (GU Serie Generale n.204 del 01-09-2023) (2023).

[18] Navarro-Aguilar, M. E., Trenc-Español, P., Pérez-Pañart, I., Gay-Sanz, B., Aladrén-Pérez, B., & Navarro-Calzada, J. (2024). Knowledge about chemsex and HIV pre-exposure prophylaxis (PREP) in emergency departments in Aragon. *Emergencias*. 10.55633/s3me/060.2024 10.55633/s3me/060.2024 39234839

[19] Jaiswal, J., Dunlap, K., Griffin, M., Cox, A., Singer, N. S, Hascher, K., & Mumba, M. (2021). Pre-exposure prophylaxis awareness, acceptability and potential stigma among medical and non-medical clinic staff in methadone treatment settings in northern New Jersey: The key role of non-medical staff in enhancing HIV prevention. *Journal of Substance Abuse Treatment*, *129*, 108371. 10.1016/j.jsat.2021.108371 34080542 10.1016/j.jsat.2021.108371 PMC8496743

[20] Krakower, D., & Mayer, K. H. (2012). Engaging healthcare providers to implement HIV pre-exposure prophylaxis. *Current Opinion in HIV and AIDS*, *7*(6), 593–599. 10.1097/COH.0b013e3283590446 23032736 10.1097/COH.0b013e3283590446 PMC3769645

[21] Blumenthal, J., Jain, S., Krakower, D., Sun, X., Young, J., Mayer, K., & Haubrich, R. (2015). Knowledge is Power! Increased provider knowledge scores regarding pre-exposure Prophylaxis (PrEP) are Associated with higher rates of PrEP prescription and future intent to prescribe PrEP. *AIDS and Behavior*, *19*(5), 802–810. 10.1007/s10461-015-0996-z 25616837 10.1007/s10461-015-0996-z PMC4417031

[22] Ogundare, M. O., Allan, F., Desai, A. P., Dirajlal-Fargo, S., Minich, N. M., & Gripshover, B. M. (2023). Integrating HIV Pre-Exposure Prophylaxis (PrEP) Education During Medical Residency: Training Outcomes and Suggestions for Learning Effectiveness. *Journal of Primary Care & Community Health*, *14*. 10.1177/21501319231207313 10.1177/21501319231207313 PMC10631327 37933559

[23] Cooper, R. L., Tabatabai, M., Juarez, P. D., Ramesh, A., Morris, M. C., Brown, K.Y.,… Juarez, P.-M. (2021). Pre-Exposure Prophylaxis Training among Medical Schools in the United States. *Journal of Primary Care & Community Health*, *12*. 10.1177/21501327211028713. 10.1177/21501327211028713 PMC8255559 34219508

[24] Cannon, S. M., Graber, S., King, H. L., Hanashiro, M., Averbach, S., Moore, D. J., & Blumenthal, J. (2021). PrEP University: A Multi-disciplinary University-based HIV Prevention Education Program. *Journal of Community Health*, *46*(6), 1213–1220. 10.1007/s10900-021-01007-x 34106369 10.1007/s10900-021-01007-x PMC8595182

[25] King, H. L., Park, E., Blanchard, H., Alvarez, K. S., Harms, M., & Broker, P. (2023). Implementation of sexual Health Curriculum Series: Educational Initiative to increase STI screening and treatment in Dallas, Texas. *Journal of Community Health*, *48*(5), 793–797. 10.1007/s10900-023-01224-6 37119350 10.1007/s10900-023-01224-6 PMC10147994

[26] Estcourt, C. S., MacDonald, J., Saunders, J., Nandwani, R., Young, I., Frankis, J.,… Flowers, P. (2023). Improving HIV pre-exposure prophylaxis (PrEP) uptake and initiation:process evaluation and recommendation development from a national PrEP program†. *Sexual Health*, *20*(4), 282–295. 10.1071/SH22170 10.1071/SH22170 37603534

[27] Herrera, M. C., Mahajan, A., Bonett, S., Aronowitz, S., Bauermeister, J., & da Teixeira, D. (2024). Facilitators and barriers to implementing HIV Testing and Pre-exposure Prophylaxis in Substance Use Treatment Programs: Perspectives of non-medical staff. *Substance use & Addiction Journal*, *45*(4), 548–558. 10.1177/29767342241274077 39234640 10.1177/29767342241274077 PMC11970037

[28] Mayer, K. H., Agwu, A., & Malebranche, D. (2020). Barriers to the wider use of pre-exposure Prophylaxis in the United States: A narrative review. *Advances in Therapy*, *37*(5), 1778–1811. 10.1007/s12325-020-01295-0 32232664 10.1007/s12325-020-01295-0 PMC7467490

